# Circulating MicroRNAs as Biomarkers for Prostate Cancer Detection and Metastasis Development Prediction

**DOI:** 10.3389/fonc.2019.00900

**Published:** 2019-09-11

**Authors:** David Bidarra, Vera Constâncio, Daniela Barros-Silva, João Ramalho-Carvalho, Catarina Moreira-Barbosa, Luís Antunes, Joaquina Maurício, Jorge Oliveira, Rui Henrique, Carmen Jerónimo

**Affiliations:** ^1^Cancer Biology and Epigenetics Group, IPO Porto Research Center (CI-IPOP), Portuguese Oncology Institute of Porto (IPO Porto), Porto, Portugal; ^2^Master in Oncology, Institute of Biomedical Sciences Abel Salazar-University of Porto (ICBAS-UP), Porto, Portugal; ^3^Department of Epidemiology, Portuguese Oncology Institute of Porto (IPO Porto), Porto, Portugal; ^4^Urology Clinic, Portuguese Oncology Institute of Porto (IPO Porto), Porto, Portugal; ^5^Department of Pathology, Portuguese Oncology Institute of Porto, Porto, Portugal; ^6^Department of Pathology and Molecular Immunology, Institute of Biomedical Sciences Abel Salazar-University of Porto (ICBAS-UP), Porto, Portugal

**Keywords:** prostate cancer, biomarkers, liquid biopsies, circulating microRNAs, miR-182-5p, miR-375-3p, detection, metastasis

## Abstract

Prostate Cancer (PCa) overdiagnosis and overtreatment, as a consequence of the limited specificity of current detection and prognostication methods, remains a major challenge in clinical practice. Therefore, development and validation of new molecular biomarkers amenable of detecting clinically significant disease is crucial. MicroRNAs (miRNA) deregulation is common in cancer, constituting potential non-invasive biomarkers for PCa detection and prognostication. Herein, we evaluated the screening and prognostic biomarker potential of two onco-microRNAs (miR-182-5p and miR-375-3p) in liquid biopsies (plasma) of PCa patients with clinically localized disease undergoing curative-intent treatment. A first cohort of 98 PCa and 15 normal prostates were used to assess PCa-specificity of miR-182-5p in tissues. A cohort composed of PCa 252 patients and 52 asymptomatic controls allowed for assessment of diagnostic and prognostic value in plasmas. After RNA extraction from tissue and plasma samples, cDNA synthesis specific for miRNAs was performed followed by measurement of miR-182-5p and miR-375-3p relative expression by RT-qPCR, using U6 snRNA gene as reference. MiR-182-5p was significantly overexpressed in PCa tissues (*p* < 0.0001) and in plasma of PCa patients (*p* = 0.0020), compared to respective controls. Moreover, miR-182-5p expression identified PCa with AUC = 0.81 (95% CI: 0.725–0.892, *p* = 0.0001) in tissue and with 77% specificity and 99% NPV (AUC = 0.64, 95% CI: 0.561–0.709, *p* = 0.0021) in plasma. Both circulating miR-182-5p and miR-375-3p levels associated with more advanced pathologic stage and the former was significantly higher in patients that developed metastasis (*p* = 0.0145). Indeed, at the time of diagnosis, circulating miR-375-3p levels predicted which patients would develop metastasis, with almost 50% sensitivity, 76% specificity, and a NPV of 89% (AUC = 0.62, 95% CI: 0.529–0.713, *p* = 0.0149). We conclude that these two circulating miRNAs might be clinical useful as non-invasive biomarkers for detection and prediction of metastasis development at the diagnosis together with clinical variables used in routine practice.

## Introduction

Prostate cancer (PCa) is the second most incident malignancy and the fifth leading cause of cancer-related death in men, worldwide, with an estimated 1.3 million new cases diagnosed and 358,989 deaths just in 2018 (1). PCa is a very heterogeneous disease, entailing quite different outcomes, from clinically indolent to lethally aggressive (2). Currently, the two most widely used PCa screening tools are serum prostate-specific antigen (PSA) levels and digital rectal examination (DRE). Serum PSA has facilitated the detection of PCa at early disease stages but owing to the lack of cancer-specificity, its use is associated with high false-positive rate and detection of non-life threatening PCa (overdiagnosis) and consequent overtreatment, associated with unnecessary patient burden and healthcare cost (3). Treatment selection for each patient relies on the combination of clinical stage, serum PSA levels and Gleason score, but inaccuracies are relatively common since patients sharing the same clinical conditions may achieve different outcomes (4). Therefore, effective screening and prognostic biomarkers for management of PCa patients remain an unmet need.

MicroRNAs (miRNAs) are small non-coding RNA molecules, with about 22 nucleotides, which are able to suppress gene expression at translation level by directly targeting mRNA molecules (4). Thus, miRNAs have been virtually linked to all biochemical processes including cancer development, and it is estimated that up to 60% of protein-coding genes' expression may be regulated by miRNA activity (5–7). MiRNA expression signatures have been shown to differ between cancer and normal tissues and also among cancer subtypes (5). Hence, these have progressively emerged as stable cancer-specific biomarkers that may help perfect diagnosis, prognostication and prediction of response to treatment. A decade ago, miRNAs were found to circulate in biological fluids, including blood with remarkable stability, thereby broadening their potential both as tumor-specific and fluid-circulating biomarkers (8).

Considering the potential of miRNAs as cancer biomarkers, several studies have contributed for the identification of the most relevant miRNAs involved in PCa biology, establishing a PCa-specific miRNA expression profile (9–13). We have previously shown that 17 miRNAs were overexpressed in PCa tissue compared to morphologically normal prostate tissue (MNPT) by microarray analysis (14). Moreover, miR-182-5p and miR-375-3p overexpression in PCa was further validated in tissue samples of two different cohorts of 80 and 114 PCa patients, respectively (14). Herein, we sought to extend those observations, analyzing miR-182-5p expression in a larger series of PCa tissues and examining the potential of circulating miR-182-5p and miR-375-3p levels as non-invasive screening and prognostic biomarkers for PCa.

## Materials and Methods

### Patients and Sample Collection

A total of 98 patients harboring PCa and submitted to radical prostatectomy at Portuguese Oncology Institute of Porto between 2001 and 2012, were recruited for our first cohort (Cohort #1). Immediately after surgery, prostate specimens were dissected for routine collection of fragments for histopathological assessment and systematic sampling for research purposes (snap-frozen and stored at −80°C). MNPTs were collected from 15 bladder cancer patients submitted to cystoprostatectomy due to bladder cancer and were used as negative controls after confirmation of the absence of prostate malignancy. All frozen tissues were cut in a cryostat for identification of target cells in hematoxylin-eosin stained slides. Then serial sectioning was performed for nucleic acid extraction, after trimming of the fragment to increase the yield (>70%) of target cells.

Additionally, plasma samples from 252 patients diagnosed with PCa at Portuguese Oncology Institute of Porto, Portugal, collected before curative-intent treatment, between 2000 and 2012 were obtained (Cohort #2). For control purposes, plasma samples were collected from 52 asymptomatic blood donors from 2009 to 2010, at the same institution. After collection of peripheral blood into EDTA-containing tubes, plasma was obtained by centrifugation at 2,000 rpm for 10 min at 4°C, and subsequently stored at −80°C until further use.

Each PCa case was staged by an uropathologist (RH) (15), and histologic grade group was determined according with Epstein classification (16). Relevant clinical data was retrieved from clinical records for both patient cohorts. Biochemical relapse was considered when patients presented two consecutive risings of serum PSA levels ≥0.2 ng/mL after surgery or 2 ng/mL above the PSA nadir after radiotherapy.

This study was approved by the institutional review board (Comissão de Ética para a Saúde) of Portuguese Oncology Institute of Porto, Portugal (IRB-CES-IPOFG-EPE 120/015). Informed consent was obtained from all patients and asymptomatic donors.

### RNA Extraction From Tissue and Plasma

RNA from tissue and circulating RNA from plasma were obtained using Triple Xtractor Reagent (GRisP, Porto, Portugal) and miRNeasy Serum/Plasma Kit (Qiagen, Hilden, Germany), respectively, according to manufacturer's instructions. RNA concentration and purity were subsequently measured in a Nanodrop ND-1000 spectrophotometer (NanoDrop Technologies).

### cDNA Synthesis

MicroRNA-specific cDNA synthesis of 100 ng of RNA in a volume of 5 μL was performed using TaqMan microRNA Reverse Transcription Kit (Applied Biosystems, Foster City, CA, USA) and Veriti® Thermal Cycler (Applied Biosystems), following manufacturer's protocol. A 1:2 dilution with DNase/RNase-Free Water (GIBCO) was performed after cDNA synthesis.

### miRNA Expression Analysis

Quantitative Real-Time PCR (RT-qPCR) was performed using specific TaqMan microRNA assays for miR-182-5p (Assay ID 000597, Applied Biosystems) and miR-375-3*p* (Assay ID 000564, Applied Biosystems), and NZYSpeedy qPCR Probe (NZYTech, Lisbon, Portugal) in a LightCycler 480 Instrument (Roche Diagnostics, Manheim, Germany) according to recommended protocol. Triplicates were performed for each sample and miRNA relative expression level was calculated by using comparative Ct method with U6 snRNA (Assay ID 001973) standing for as a reference gene. Relative expression was calculated under the following formula:

$$\begin{array}{l} Relative\;miRNA\;expression=2^{-\left(Ct\left(miRNA\right)-Ct\left(U6\;snRNA\;\right)\right)} \end{array}$$

### Statistical Analysis

Kruskal-Wallis and Mann-Whitney non-parametric tests were used to evaluate differences in miRNAs expression levels and associations between miRNA expression and clinical variables.

For each miRNA, receiver operator characteristics (ROC) curves were constructed by plotting the true positive (sensitivity) against the false-positive (1-specificity) rate, and Area Under Curve (AUC) was calculated. Specificity, sensitivity, positive predictive value (PPV), negative predictive value (NPV), and accuracy were determined for each miRNA by applying cut-off values that were established based on the highest value obtained in ROC curve analysis according to Youden's J index (17, 18). Diagnostic biomarker performance was calculated having in consideration a 5 year PCa prevalence in Portuguese population of 1122.5/100000 habitants (19).

Kaplan-Meier curves were constructed, and log-rank test was used to compare metastasis free survival (MFS) between groups considering clinicopathological variables (pathological stage, Grade Group) and categorized miRs expression levels status (*P* < 50; low expression levels and *P* ≥ 50; high expression levels). MFS was calculated as the time between the first day of treatment and the day of first imaging exam showing metastasis. Cox proportional hazards regression was employed to calculate hazard ratios (HR) and 95% confidence intervals (CI). A backwards Wald multivariable Cox-regression model comprising all significant variables on univariable analysis was computed to determine whether miRs expression levels were independently associated with MFS.

Statistical analyses were performed using SPSS 25.0 software for Mac (IBM-SPSS Inc., Chicago, IL, USA) and GraphPad Prism 7.0 software for Mac (GraphPad Software Inc., La Jolla, CA, USA). A result was considered statistically significant when *p* < 0.05.

## Results

### Cohort #1

PCa tissues were obtained from 98 patients submitted to radical prostatectomy. Forty-three patients were stage pT2, 37 patients were pT3a, and 18 patients were pT3b. No significant differences in median age between patients and controls was observed. The median time of patients' follow-up was 134 months, and during this period, 53 patients developed biochemical relapse and among those, 16 developed imagological documented metastases (Table 1).

**Table 1:** Clinical and pathological data of morphologically normal prostate and prostate cancer patients (Cohort #1).

**Clinicopathological variables**	**Tissue samples**	
	**MNPT**	**PCa**
Patients, *n*	15	98
Median age, *years* (range)	64 (45–80)	63 (46–73)
Median PSA (*ng/mL*) (range)	n.a.	8.69 (2.40–21)
**Pathological stage (pT)**, ***n*** **(%)**		
pT2	n.a.	43 (43.9%)
pT3a	n.a.	37 (37.8%)
pT3b	n.a.	18 (18.3%)
**Grade group**, ***n*** **(%)**		
1	n.a.	26 (26.5%)
2	n.a.	28 (28.6%)
3	n.a.	27 (27.5%)
4	n.a.	4 (4.1%)
5	n.a.	13 (13.3%)
**Follow up**		
Median, *Months* (range)	n.a.	134 (51–203)
Biochemical recurrence, *n* (%)	n.a.	53 (54.1%)
Metastasis, *n* (%)	n.a.	16 (16.3%)
Death, *n* (%)	n.a.	8 (8.16%)

*MNTP, Morphologically Normal Prostatic Tissue; PCa, Prostate Cancer; n.a., not applicable*.

**Figure 1:**
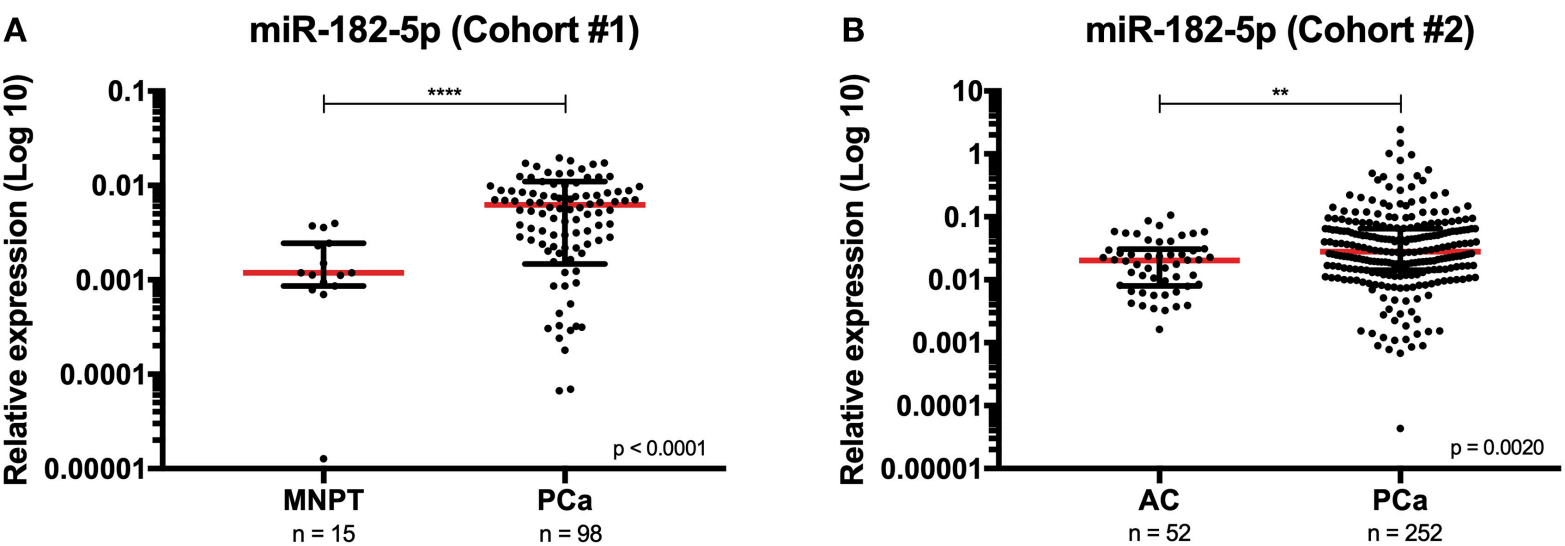
Distribution of miR-182-5p expression levels in Cohort #1 [morphologically normal prostate (MNPT) and prostate cancer (PCa) tissues] **(A)**, and in Cohort #2 [asymptomatic controls (AC) and PCa patients] **(B)**. Mann-Whitney *U*, ****p < 0.0001 and **p <0.01. Red horizontal line represents the relative expression levels' median.

Quantitative analysis disclosed that miR-182-5p was significantly overexpressed in PCa tissue compared to control samples (*p* < 0.0001) (Figure 1A). Furthermore, relative expression of this microRNA discriminated between malignant and non-malignant prostate tissue with 60.20% sensitivity, 100% specificity and PPV, and 99.55% NPV, corresponding to an AUC of 0.81 (95% CI: 0.725–0.892, *p* = 0.0001), and 65.49% accuracy (Supplementary Figure 1A).

### Cohort #2

Because our main goal was to explore the screening and prognostic biomarker potential of miR-182-5p and miR-375-3p in liquid biopsies, expression of these two miRNAs was evaluated in plasma samples of a cohort of 252 PCa and 52 asymptomatic controls (AC). Detailed clinical and pathological data is depicted in Table 2. Although, the median age of patients significantly differed from asymptomatic controls (62 vs. 58 years, *p* < 0.0001), no significant correlation was found between age and miRNAs expression levels. Regarding pathological stage, 83 patients were classified as pT2, 127 patients as pT3a, and 35 patients as pT3b stage. Median patient follow-up time was 93 months, and during this period 112 patients developed biochemical relapse and 40 developed metastases. All patients were treated with curative intent (specifically, 245 patients were submitted to radical prostatectomy and 7 were treated with external beam radiotherapy).

**Table 2:** Clinical and pathological data of asymptomatic controls and prostate cancer patients (Cohort #2).

**Clinicopathological variables**	**Plasma samples**	
	**AC**	**PCa**
Patients, *n*	52	252
Median age, *years* (range)	58 (54–64)	62 (46–76)
Median PSA (*ng/mL*) (range)	n.a.	8.4 (0.68–837)
**Pathological stage (pT)**, ***n*** (%)^*^		
pT2	n.a.	83 (32.9%)
pT3a	n.a.	127 (50.4%)
pT3b	n.a.	35 (13.9%)
**Grade group**, ***n*** **(%)**		
1	n.a.	48 (19.0%)
2	n.a.	94 (37.3%)
3	n.a.	68 (27.0%)
4	n.a.	15 (5.9%)
5	n.a.	27 (10.8%)
**Follow up**		
Median, *Months* (range)	n.a.	93 (5–216)
Biochemical recurrence, *n* (%)	n.a.	112 (44.4%)
Metastasis, *n* (%)	n.a.	40 (15.9%)
Death, *n* (%)	n.a.	16 (6.3%)

*AC, Asymptomatic Controls; PCa, Prostate Cancer; n.a., not applicable*.

* *For 7 patients treated with RT, clinical stage was considered. From those, 3 patients were cT3a (1.2%) and 4 were cT3b (1.6%)*.

Paralleling the results observed in tissue samples, circulating miR-182-5p expression was also significantly increased in PCa patients compared to controls (*p* = 0.0020) (Figure 1B). Furthermore, circulating miR-182-5p discriminated PCa samples from controls with 47.62% sensitivity, 76.92% specificity, and 99.23% NPV, corresponding to an AUC of 0.64 (95% CI: 0.561–0.709, *p* = 0.0021) (Supplementary Figure 1B). No statistically significant differences were disclosed for circulating miR-375-3p expression levels between patients and controls.

**Figure 2:**
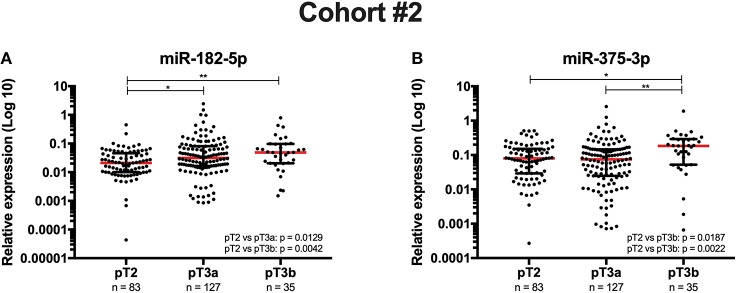
Circulating miR-182-5p's **(A)** and miR-375-3p's **(B)** expression levels at diagnosis according to pathological stage (Cohort #2). Kruskal-Wallis, **p < 0.01 and *p < 0.05. Red horizontal line represents the relative expression levels' median.

**Figure 3:**
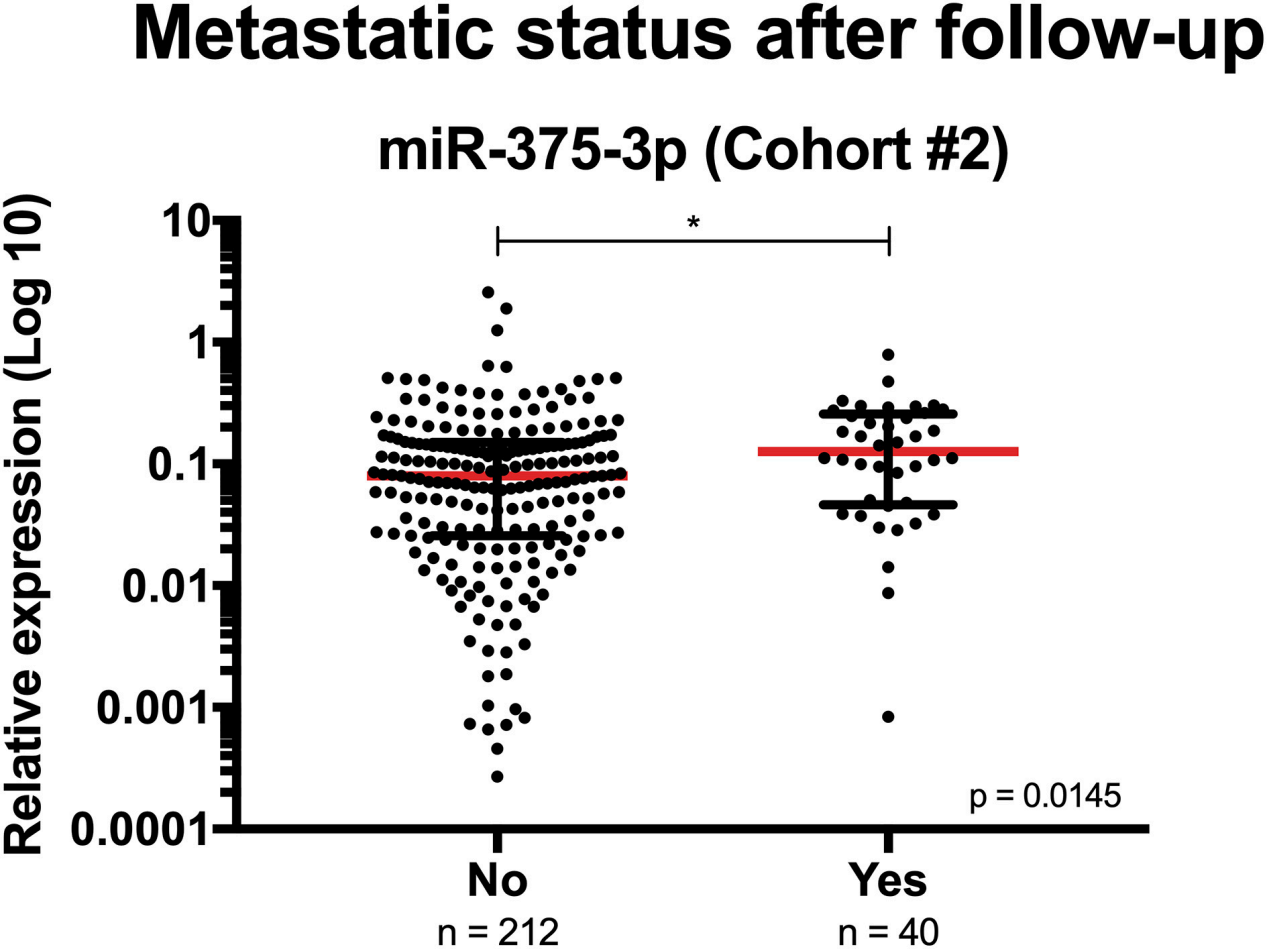
Circulating miR-375-3p's expression levels at diagnosis according to metastatic status after follow-up (Cohort #2). Mann-Whitney *U*, *p < 0.05. Red horizontal line represents the relative expression levels' median.

Concerning clinicopathologic correlates, although no significant associations were found at diagnosis between circulating miRNAs expression and serum PSA levels or grade group, higher circulating levels of both miR-182-5p (pT2 vs. pT3a *p* = 0.0129; pT2 vs. pT3b *p* = 0.0042) and miR-375-3p (pT2 vs. pT3a *p* = 0.0187; pT3a vs. pT3b *p* = 0.0022) associated with advanced pathologic stage (Figure 2). Furthermore, at diagnosis, circulating miR-375-3p was significantly higher in PCa patients that developed metastasis comparing with those that did not metastasized (*p* = 0.0145) (Figure 3). Indeed, higher circulating miR-375-3*p* expression levels predicted development of metastasis during follow-up with 48.72% sensitivity, 75.59% specificity, and 88.95% NPV, corresponding to an AUC of 0.62 (95% CI: 0.529–0.713, *p* = 0.0149), and 71.43% accuracy (Table 3 and Supplementary Figure 2). The same was not observed for circulating miR-182-5p expression levels or serum PSA levels.

**Table 3:** Metastasis prediction performance of miR-375-3p's circulating levels (Cohort #2).

**miR-375-3p**	**Sensitivity %**	**Specificity %**	**Positive predictive value %**	**Negative predictive value %**	**Accuracy %**
Plasma	48.72	75.59	26.76	88.95	71.43

**Figure 4:**
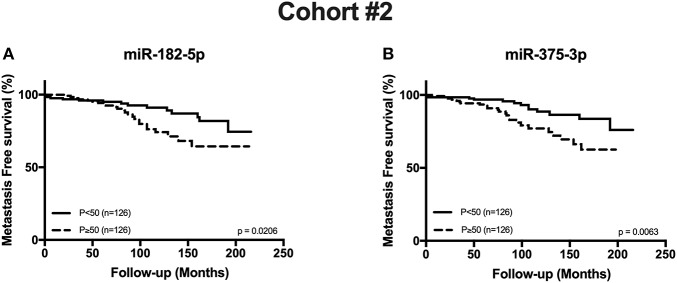
Metastasis Free Survival curves in Cohort #2 according to miR-182-5p **(A)** and miR-375-3p **(B)** expression levels.

MFS was significantly reduced in patients with increasing grade group and pT stage (both *p* < 0.0001), as expected (Supplementary Figure 3). Interestingly, high circulating levels (*P* ≥ 50) of both miR-182-5p and miR-375-3p significantly associated with reduced MFS (*p* = 0.0206 and *p* = 0.0063, respectively) (Figure 4). Moreover, when GG2 and pT3a patients were cumulatively selected, higher circulating miR-375-3p levels also associated with decreased MFS (Supplementary Figure 4). Multivariable Cox-regression analysis demonstrated that grade group (GG1 vs. GG4-5), pathologic stage (pT2 vs. pT3a and pT2 vs. pT3b) and circulating miR-375-3p levels independently predicted MFS (Table 4). Higher circulating miR-182-5p levels (*p* = 0.0274) only significantly associated with MFS in univariable analysis (Supplementary Table 1). Due to lack of events, disease specific survival analysis was not performed.

**Table 4:** Cox regression models assessing the potential of clinical variables and circulating miRs levels in the prediction of metastasis free survival (Cohort #2).

**Metastasis free survival**	**Variable**	**HR**	**95% CI for HR**	***P* value**
Multivariable	**Grade group (GG)**			
	GG1 vs. GG2	2.208	0.438–11.136	0.3373
	GG1 vs. GG3	4.766	0.993–22.869	0.0510
	GG1 vs. GG4–5	8.779	1.787–43.138	**0.0075**
	**Pathological stage (pT)**			
	pT2 vs. pT3a	16.233	2.100–125.490	**0.0076**
	pT2 vs. pT3b	20.383	2.396–173.399	**0.0058**
	**miR-375-3p**			
	*P* < 50 vs. *P* ≥ 50	2.153	1.039–4.461	**0.0392**

*HR, Hazard Ratio*.

*Bold values represent statistically significant p-values*.

## Discussion

Since their first report in the context of malignant disease in 2002 (20), miRNAs have shown great potential as cancer biomarkers. In particular, miRNAs circulating in body fluids, such as plasma, have been consistently described since 2008 (4, 21) and have broadened the biomarker spectrum of miRNAs as non- or minimally-invasive tools for cancer diagnosis, prognostication and disease monitoring (22). In that vein, we tested the screening and prognostic performance of circulating miR-182-5p and miR-375-3p in PCa patients.

Using microarray analysis, we previously found a set of 17 miRNAs overexpressed in PCa tissues compared to MNPT, among which miR-182-5p and miR-375-3p were validated in a larger series of PCa tissues (*n* = 80 and *n* = 114, respectively) vs. MNPT (*n* = 15) (14). Because the number of samples used to validate miR-182-5p was relatively limited, we extended it to 98 in the present series. We confirmed that miR-182-5p was significantly overexpressed in PCa tissues compared to non-neoplastic prostate tissue, further supporting its proposed oncogenic role in prostate carcinogenesis (23–25). Furthermore, miR-182-5p expression levels alone discriminated tumorous from non-tumorous prostate tissue with 100% Specificity and PPV and 99.55% NPV, with an AUC of 0.81, which is superior to that reported previously by Schaefer et al. that achieved an AUC of 0.70 (95% CI: 0.62–0.79) comparing 76 matched PCa and adjacent normal tissues (23).

Having confirmed the overexpression of both miRNAs in tumor tissues, our major goal was then to assess their clinical utility in liquid biopsies, as tissue samples may not fully represent tumor heterogeneity (22) and can be more easily used as a source for non-invasive early detection, diagnosis or prognostication of PCa. Interestingly, circulating miR-182-5p, but not miR-375-3p, was also found overexpressed in PCa patients compared to asymptomatic controls, being able to identify PCa with 76.92% specificity and 99.23% NPV, although sensitivity was modest. Remarkably, our results indicate that circulating miR-182-5p performance for PCa detection is similar to that of urinary PCA3, outperforming serum PSA (26). Thus, although single circulating miR-182-5p analysis might be a suboptimal early detection test alone due to its modest sensitivity, it might complement other routinely available tests, increasing the specificity of serum PSA testing, potentially reducing the number of unnecessary biopsies.

Although, several miRNAs have shown promise as PCa screening and diagnostic biomarkers, differences in cohort sizes, patient heterogeneity and sample sources, have prevented more thorough validation (4). Thus far, in plasma samples, the only consistently reported miRNA with better diagnostic performance than miR-182-5p is miR-21, disclosing AUC of 0.799–0.877, although the larger cohort published only comprised 57 PCa patients (27, 28). Recently, another study, which included 100 PCa and 50 control plasma samples, demonstrated that addition of miR-21 to Prostate Health Index (PHI) significantly increased the sensitivity to 95.5% with 100% a specificity for detecting patients with localized PCa (29). Nonetheless, our study is the first to explore the potential of miR-182-5p as PCa detection biomarker, although validation in a larger independent cohort is required to fully demonstrate its clinical usefulness, both in localized disease and metastatic settings.

Conversely, concerning miR-375-3p circulating expression levels and contrarily to previous publications (13, 28), we were not able to confirm its value as diagnostic biomarker for PCa. It should be recalled, however, that our cohort more than quadruplicates those studies (57 and 31 PCa patients), in which patients with benign prostatic hyperplasia (BPH) were used as controls, instead of asymptomatic blood donors, as in the present study. Moreover, the combination with other circulating miRs or relevant clinical variables might perfect its overall performance as reported for miR-221 and PHI (29).

Because PCa overtreatment is a major concern, it seemed pertinent to assess whether the selected miRNAs might provide independent prognostic information, identifying patients at risk for disease progression, which are those that benefit from therapeutic intervention. Interestingly, high circulating levels of both miR-182-5p and miR-375-3p were associated with more advanced pathological stages, suggesting that they might provide relevant prognostic information. Indeed, at the time of diagnosis, higher circulating miR-375-3p levels identified patients more prone to develop metastatic disease with 71.43% accuracy. However, in our cohort, serum PSA levels did not discriminate between patients that developed metastasis and those that did not. Furthermore, survival analysis disclosed that higher circulating miR-375-3p levels significantly associated with lower MFS, including in PCa patients with grade group 2 and pT3a stage. This is a particularly interesting result as the prognosis of this group of patients is difficult to ascertain (30) and miR-375-3p circulating levels, at diagnosis, seems to identify two groups with quite dissimilar outcome.

Previous studies have shown that circulating levels of miR-375-3p are higher in patients harboring disseminated or metastatic castration-resistant (CRPC) PCa, compared to those with localized disease (9, 13, 31), indicating that miR-375-3p expression increases along disease progression. Our results, however, are the first to demonstrate that miR-375-3p circulating levels senses the potential for PCa progression already at diagnosis, reflecting tumor's biological and clinical aggressiveness, even among clinically localized disease, amenable to curative-intent treatment. Because circulating miR-375-3p was shown to be an independent predictor of metastases development, it might perfect routinely used nomograms that assess PCa risk of progression, identifying patients more prone to endure metastatic disease and that may benefit from more adequate therapeutic intervention (13). On the other hand, it is tempting to speculate whether PCa with very low circulating miR-375-3p levels constitute a subpopulation with minimal risk of disease progression, amenable to conservative management, thus reducing the risk of overtreatment. Therefore, circulating miR-375-3p levels might be a helpful tool to personalized PCa treatment options, reducing healthcare costs and therapy-related side-effects, thus increasing patients' life quality.

In conclusion, our exploratory study highlights the screening and prognostic potential of circulating miR-182-5p and miR-375-3p in PCa patients, which might constitute valuable tools to improve patient management along with the currently used biomarkers, thus perfecting risk stratification and reducing overtreatment.

## Ethics Statement

This study was carried out in accordance with the recommendations of Comissão de Ética para a Saúde of Portuguese Oncology Institute of Porto, Portugal (IRB-CES-IPOFG-EPE120/015) with written informed consent from all subjects. All subjects gave written informed consent in accordance with the Declaration of Helsinki. The protocol was approved by the Comissão de Ética para a Saúde of Portuguese Oncology Institute of Porto, Portugal.

## Author Contributions

JR-C, RH, and CJ conceived and designed the experiments. DB, VC, and CM-B performed the experiments. VC, DB-S, LA, RH, and CJ analyzed the data. JM and JO selected the patients and provided the clinical data from clinical charts. All authors read and approved the final manuscript.

### Conflict of Interest Statement

The authors declare that the research was conducted in the absence of any commercial or financial relationships that could be construed as a potential conflict of interest.
