# Supplementary material for: Circulating MicroRNAs as Biomarkers for Prostate Cancer Detection and Metastasis Development Prediction
Source: Front Oncol. 2019 Sep 11;9:900. doi: 10.3389/fonc.2019.00900 (PMC6749029; doi:10.3389/fonc.2019.00900)
Supplement: Supplementary file 1 [file Data_Sheet_1.docx]

**Supplementary Table 1.** Cox regression models assessing the potential of clinical variables and circulating miRs levels in the prediction of metastasis free survival (Cohort #2).

| **Metastasis Free Survival** | **Variable** | **HR** | **95% CI for HR** | **P value** |
| --- | --- | --- | --- | --- |
| **Univariable** | **Grade Group (GG)** | | | |
|  | GG1 vs GG2 | 4.262 | 0.890-20.411 | 0.0697 |
|  | GG1 vs GG3 | 11.228 | 2.452-51.425 | **0.0018** |
|  | GG1 vs GG4-5 | 30.156 | 6.506-139.777 | **<0.001** |
|  | **PSA** | | | |
|  | <10 ng/mL vs ≥10 ng/mL | 1.535 | 0.785-3.003 | 0.2105 |
|  | **Pathological stage (pT)** | | | |
|  | pT2 vs pT3a | 25.867 | 3.494-191.507 | **0.0014** |
|  | pT2 vs pT3b | 65.477 | 8.369-512.305 | **<0.001** |
|  | **miR-182-5p** | | | |
|  | P<50 vs P≥50 | 2.068 | 1.084-3.945 | **0.0274** |
|  | **miR-375-3p** | | | |
|  | P<50 vs P≥50 | 2.668 | 1.369-5.199 | **0.0039** |

HR – Hazard Ratio

**
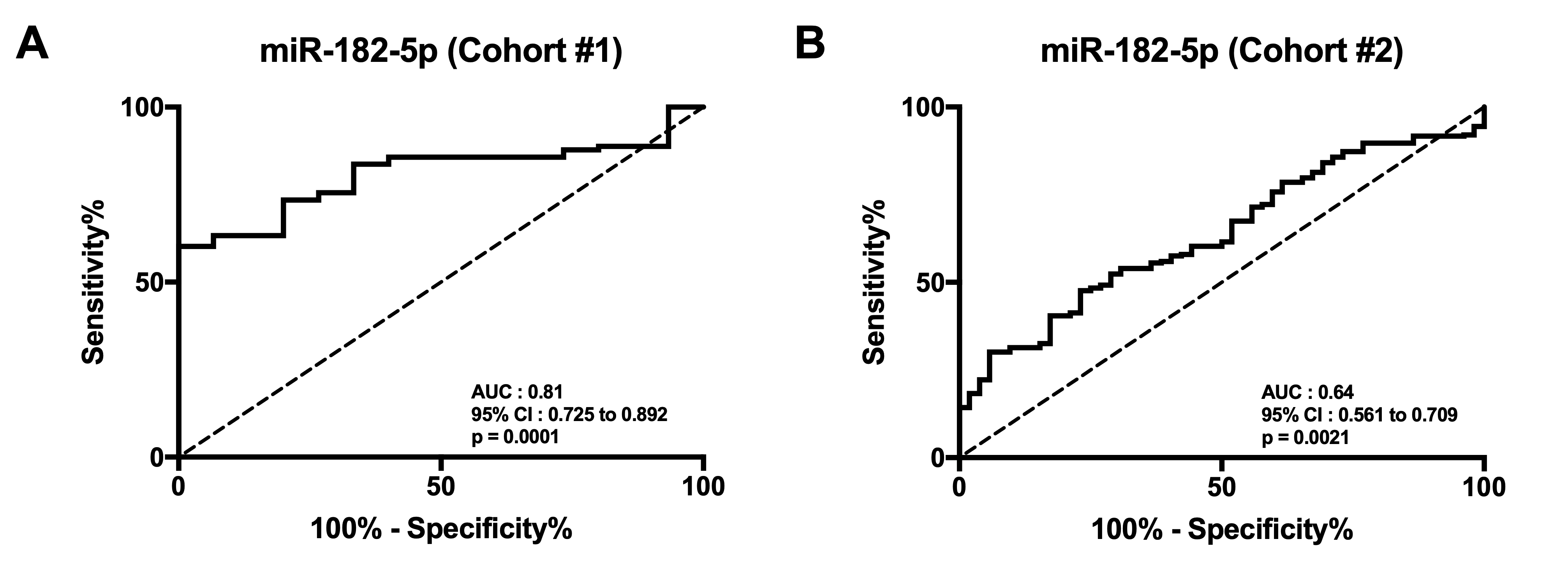
**

**Supplementary Figure 1.** Receiver operation characteristic (ROC) curves of miR-182-5p in Cohort #1 (A) and Cohort #2 (B) patients for diagnosis of PCa. Reference line is in dash and ROC curve is in solid line.

**
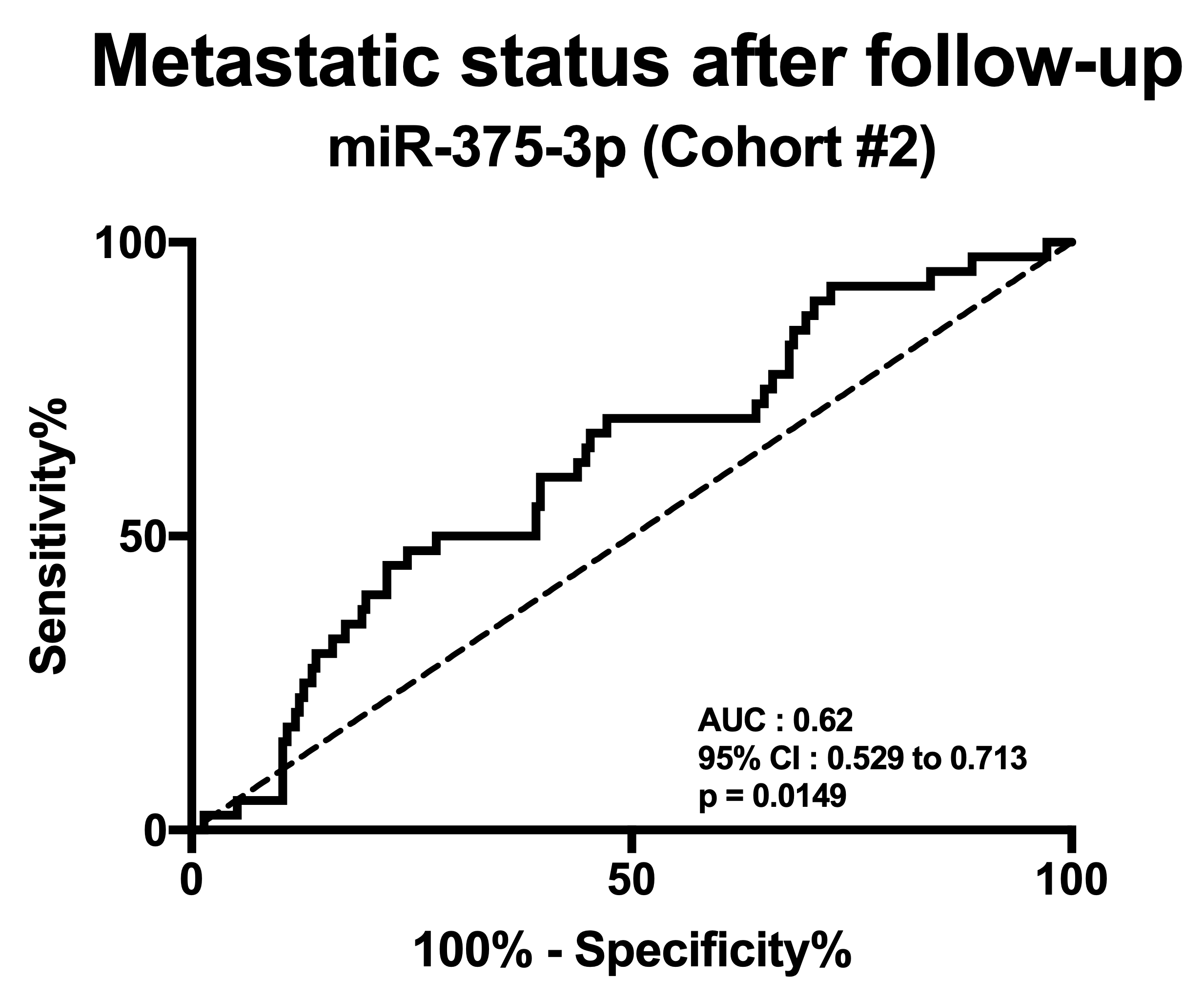
**

**Supplementary Figure 2.** Receiver operation characteristic (ROC) curve of circulating miR-375-3p for diagnosis for metastasis development prediction (Cohort #2). Reference line is in dash and ROC curve is in solid line.


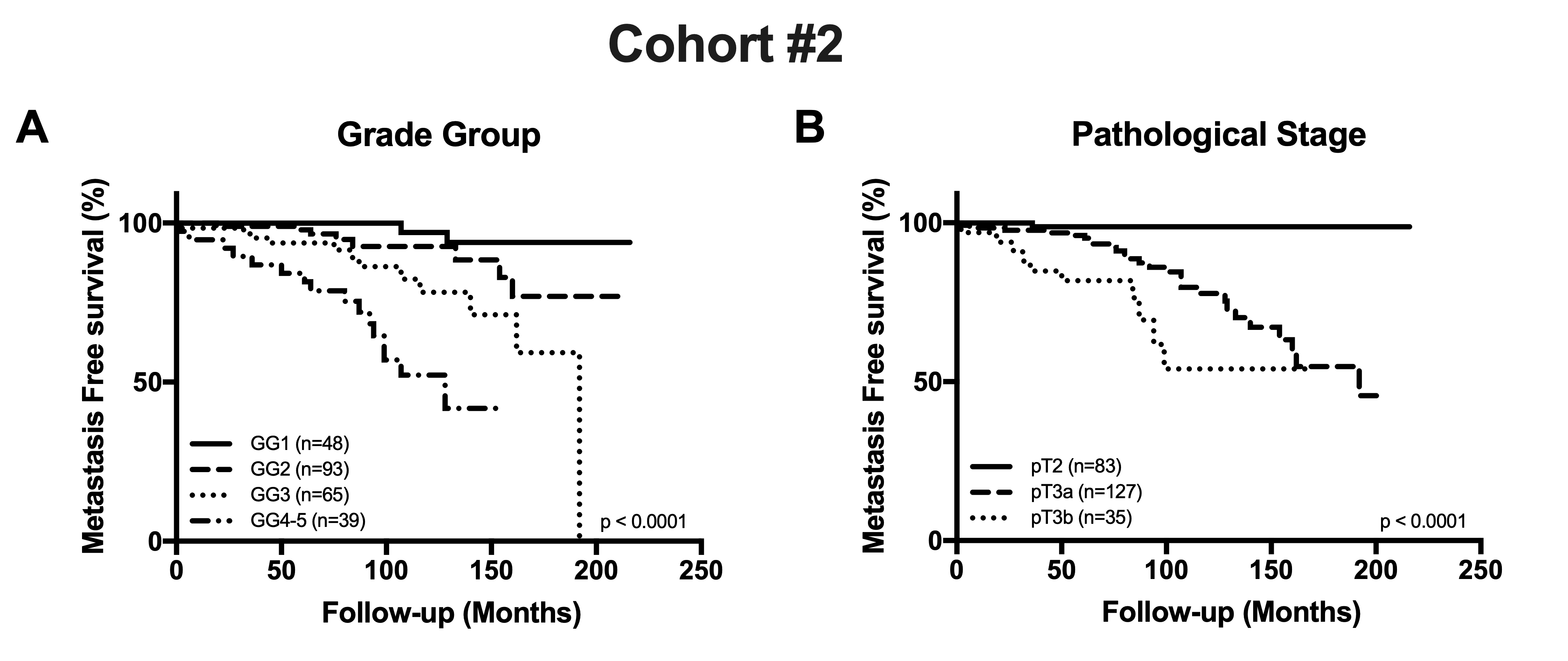
**Supplementary Figure 3.** Metastasis Free Survival curves in Cohort #2 patients according to Grade Group (A) and Pathological stage (B).


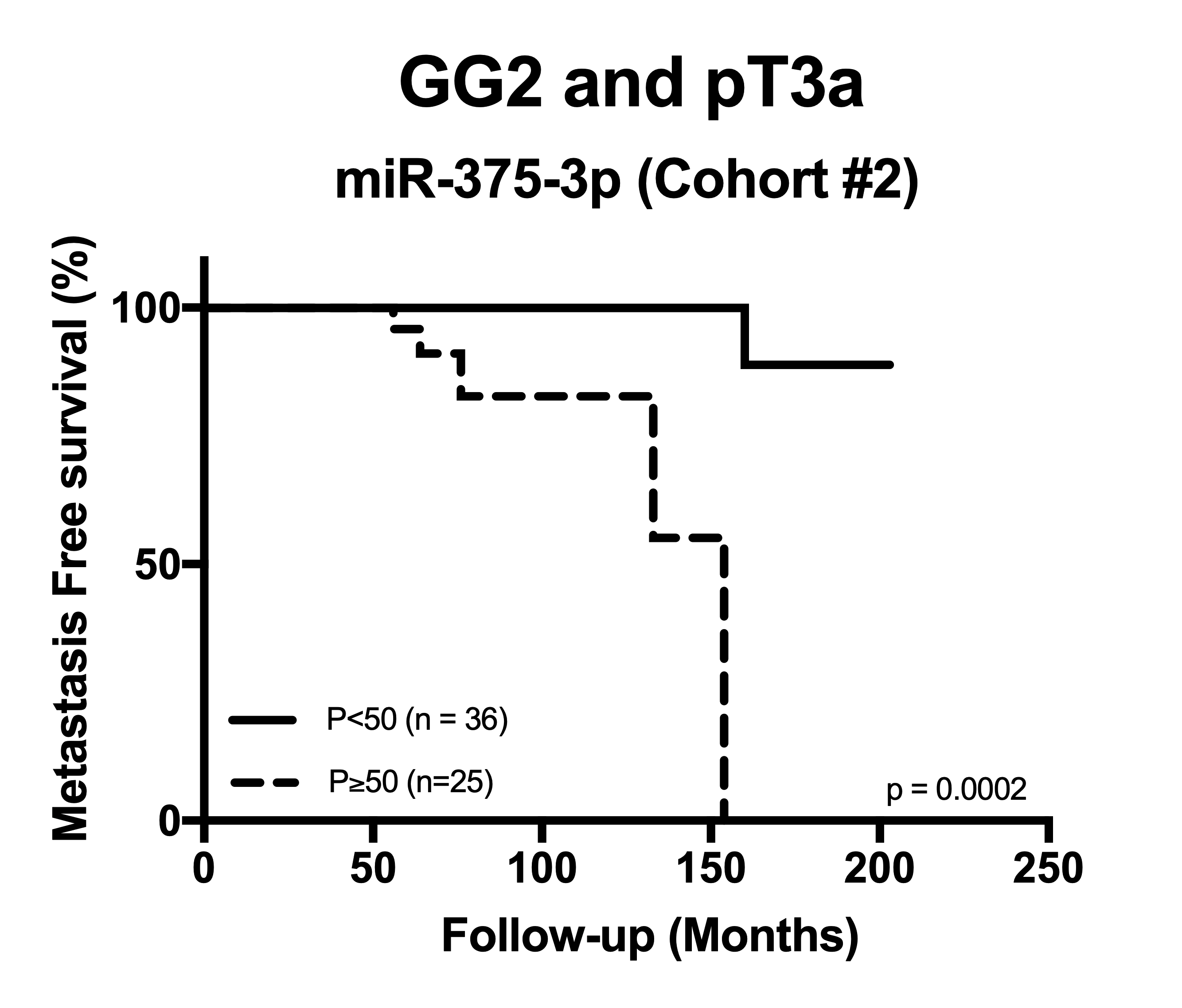


**Supplementary Figure 4.** Metastasis Free Survival curve in cohort #2 for patients with GG2 and pT3a according to circulating miR-375-3p levels at diagnosis.
